# Development and validation of a novel model for characterizing migraine outcomes within real-world data

**DOI:** 10.1186/s10194-022-01493-x

**Published:** 2022-09-21

**Authors:** Nada A. Hindiyeh, Daniel Riskin, Kimberly Alexander, Roger Cady, Steven Kymes

**Affiliations:** 1 Stanford Headache Clinic at Hoover Pavilion, Stanford, CA USA; 2 Verantos, Inc., Menlo Park, CA USA; 3 Lundbeck LLC, Deerfield, IL USA; 4 RK Consults, Ozark, MO USA; 5 Missouri State University, Springfield, MO USA

**Keywords:** Artificial intelligence, Electronic health records, Migraine, Outcome model, Real-world evidence

## Abstract

**Background:**

In disease areas with ‘soft’ outcomes (i.e., the subjective aspects of a medical condition or its management) such as migraine or depression, extraction and validation of real-world evidence (RWE) from electronic health records (EHRs) and other routinely collected data can be challenging due to how the data are collected and recorded. In this study, we aimed to define and validate a scalable framework model to measure outcomes of migraine treatment and prevention by use of artificial intelligence (AI) algorithms within EHR data.

**Methods:**

Headache specialists defined descriptive features based on routinely collected clinical data. Data elements were weighted to define a 10-point scale encompassing headache severity (1–7 points) and associated features (0–3 points). A test data set was identified, and a reference standard was manually produced by trained annotators. Automation (i.e., AI) was used to extract features from the unstructured data of patient encounters and compared to the reference standard. A threshold of 70% close agreement (within 1 point) between the automated score and the human annotator was considered to be a sufficient extraction accuracy. The accuracy of AI in identifying features used to construct the outcome model was also evaluated and success was defined as achieving an F1 score (i.e., the weighted harmonic mean of the precision and recall) of 80% in identifying encounters.

**Results:**

Using data from 2,006 encounters, 11 features were identified and included in the model; the average F1 scores for automated extraction were 92.0% for AI applied to unstructured data. The outcome model had excellent accuracy in characterizing migraine status with an exact match for 77.2% of encounters and a close match (within 1 point) for 82.2%, compared with manual extraction scores—well above the 70% match threshold set prior to the study.

**Conclusion:**

Our findings indicate the feasibility of technology-enabled models for validated determination of soft outcomes such as migraine progression using the data elements typically captured in the real-world clinical setting, providing a scalable approach to credible EHR-based clinical studies.

**Graphical Abstract:**

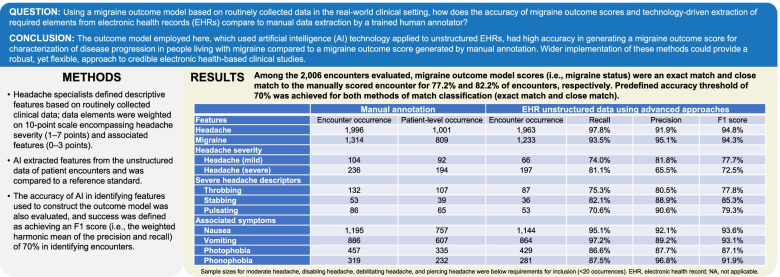

## Background

In recent years, the need to support informed decision making in treatment, payer policy, and regulation has led policy makers to broaden efficacy information they seek from randomized placebo-controlled clinical trials with limited generalizability [1] and consider a focus on real-world evidence (RWE) collected in routine clinical practice [2, 3]. The goal of this augmentation is to gain insights into the effectiveness and efficiency of clinical treatments at a population level outside of results derived in a high-resource clinical trial setting studying a stringently defined disease population. RWE is increasingly employed not only to understand real-world outcomes, but also to power studies sufficiently to enable subgroup analytics, comparative effectiveness, and tailored treatment plans [4, 5].

Evidence generation requires an understanding of outcomes, but some outcomes are more difficult to identify than others. Even within the regulated confines of a clinical trial, capturing subjective aspects of a medical condition or its management (so-called ‘soft’ outcomes) can be problematic, resulting in a reporting bias toward objective or ‘hard’ outcomes [6, 7]. In RWE, while hard outcomes such as heart attack and death are captured in claims data and death registries, soft outcomes such as worsening pain or depression are inconsistently captured within routine data [8].

Beyond the attempt to transplant outcome models directly from randomized trials and populate them with incomplete routine data, to date there has been limited work to develop and validate models to measure treatment in real-world clinical settings using soft outcomes [1]. Unfortunately, in many fields, including neurology, such a simplistic transfer is unsuccessful due to the many differences between the population of patients in a randomized trial and the real-world settings of individualized patient care [9, 10]. Typically, these include patient eligibility and selection bias, intensive trial monitoring conditions that do not reflect the frequency of routine clinic visits, placebo or nocebo effects, and the ability to implement lengthy questionnaires that are rarely utilized or fully documented in routine practice. Thus, there is a need for new, validated models to be created that account for the actual data captured in routine care that measure the patient’s condition.

In this context, an effort was undertaken to create an outcome model in neurology using the data elements typically captured in the clinical care setting. Prevention of migraine was selected because it is a highly prevalent and clinically important condition, and it presents challenges in routine data collection. In this study, we aimed to 1) define a migraine outcome model based on routinely collected data in the real-world clinical setting, 2) develop technology to extract required elements of the model, 3) evaluate the accuracy of technology-driven extraction of required elements from data contained in electronic health records (EHRs) compared with manual extraction by a trained human annotator, and 4) characterize the accuracy of a migraine outcome score based on automated extraction compared to the trained annotator.

## Methods

### Migraine outcome model development

A migraine outcome model was defined by a panel of two headache specialists (NAH and RC). To construct the model, the specialists based on their clinical experience defined elements (structured or unstructured data) likely to be captured in the EHR, that reflect the diagnosis and progression of migraine. Structured data included predetermined fields in fixed formats typically used to collect data for payment, or for regulatory or public health purposes, while unstructured data comprised the narrative written by the physician to record information used in patient management (and to maintain the medico-legal record). Although unstructured data often contain more complete information captured during a patient visit than do structured data, interpretation requires either human review and manual extraction, or sophisticated software for automated extraction.

The selected features were migraine-associated headache; headache severity (mild, moderate, severe); severity headache descriptors (including pulsating, debilitating, stabbing, throbbing, disabling, and piercing); headache progression (documented improvement or worsening); and commonly reported associated symptoms, which included photophobia, phonophobia, nausea, and vomiting.

The model was focused on symptoms since these are reflective of the patient’s migraine experience. Each selected data element was weighted to define a 10-point scale encompassing headache severity (1–7 points) and associated features (0–3 points) in a procedure consistent with current US regulatory guidance for measurement of response to acute treatment [11]. In this model, headache severity was scored as none or no headache documented (1 point); mild or severity not documented (3 points); moderate (5 points); severe or severe headache descriptor (7 points). Encounters with multiple headache features were assigned the highest headache severity represented. Associated features (nausea, vomiting, and either photophobia or phonophobia) each scored 1 point, when present.

### Technology optimization for extraction of features

#### Data source and study population

Deidentified EHR from a US tertiary care academic medical center containing information recorded between 2018 and 2020 were studied to identify primary care and neurology encounters for inclusion in the study. To increase the number of encounters representing visits focused on migraine, records were selected based on a random sampling with patient-level and encounter-level filters applied. Patient-level filters included presence of migraine in the structured or unstructured medical records and presence of at least two evaluable encounters.

For each selected patient, two evaluable encounters with at least 2 weeks of separation between the encounters were selected for the study. Evaluable encounters were those in which the primary reason for the consultation was the complaint of headache or in which there were a minimum of two mentions of headache within the encounter narrative. Patients and associated encounters were separated into training and validation data sets.

#### Reference standard creation

To optimize and validate the accuracy of automated feature extraction of data elements included in the migraine outcome model, a reference standard was created. Two independent, trained annotators with clinical degrees manually reviewed each record and labeled each feature and associated meta-data. Annotators received training on the annotation application, feature and meta-data definitions, and appropriate usage prior to review and annotation of encounters. Features included clinical concepts such as headache, migraine, nausea, vomiting, photophobia, and phonophobia. Meta-data included attributes that change the meaning for a documented feature such as negation, severity, and descriptors. Migraine features were tested at the level of each encounter, i.e., it was not assumed that the same symptoms persisted longitudinally from one encounter to the next. Thus, accuracy required that a feature be correctly identified in a specific encounter.

To ensure adequate quality in reference standard creation, annotators were blinded to each other’s annotation and inter-annotator agreement was measured daily by Cohen’s kappa score. A minimum kappa score of 0.7 was required for the reference standard to be considered adequate. After kappa score calculation, all cases of disagreement were reviewed by both annotators for resolution. Unresolved cases were escalated to a third annotator for resolution.

#### Automated feature extraction

This study included the deployment of natural language processing (NLP) and machine learning algorithms, both aspects of artificial intelligence (AI). These were applied to extract features from the unstructured data of filter-enriched encounters. For example, NLP may identify the features headache, nausea, and vomiting in different parts of an encounter narrative. Machine-learned associations may identify patterns supporting the disambiguation of abbreviations such as “MA” to “migraine with aura” instead of “mass,” “medical assistant,” or “Massachusetts.” NLP architecture and pipeline employed has been previously described [12].

Both the NLP and inference rules were optimized to extract for the clinical domain area by Verantos, Inc. (Menlo Park, CA). Since structured data are often used to identify clinical concepts in RWE, features were separately extracted from data in the EHRs using structured query language (SQL) to provide a comparison for accuracy of feature extraction from unstructured data.

### Statistical analyses

The primary study objective was to evaluate the accuracy of automated scoring of migraine severity from elements extracted from the EHR compared with manual scoring. Accuracy determination for the migraine outcome model was performed using R programming language, version 3.3.2. Results were reported as the percentage of encounters with matching migraine outcome scores based on automated versus manual feature extraction. Matches were defined as ‘exact’ (matching the manual reference score exactly on the 10-point scale) or ‘close’ (matching the manual reference score within 1 point on the 10-point scale). For this study, success was defined as achieving a close match in migraine outcome score among at least 70% of encounters.

We also evaluated the accuracy of automated feature extraction, as that was critical to automated scoring. Therefore, each element of the outcome model was compared against the manual reference standard in terms of recall, precision, and F1 score. Recall (sensitivity) was defined as the percent of data elements identified by manual annotation that were also identified through automated annotation. Precision (positive predictive value) was defined as the percent of data elements identified through automated annotation that were also identified by manual annotation. The F1 score was calculated as the weighted harmonic mean of the precision and recall. For this study, an average F1 score threshold was set at 80% to demonstrate sufficient accuracy of automated feature extraction. Concepts with fewer than 20 occurrences were excluded from accuracy measurements. The average accuracy measures were weighted on reference standard occurrence counts to account for variability in feature occurrence among encounters. Microsoft Excel 365 was used for all data analyses.

## Results

Accuracy of automated feature extraction of data elements included in the migraine outcome model was evaluated in 2,006 encounters from 1,003 patients. By manual annotation, encounter-level feature occurrence ranged from < 20 for ‘piercing headache’ to 1,996 for ‘headache.’ Only data elements with at least 20 occurrences were included in the migraine outcome model; features (such as ‘piercing headache’) that occurred in fewer than 20 encounters were excluded.

Table 1 shows results from the 11 data elements, each with a sample size ≥ 20, included in the model. The average F1 scores for these features were 92.0% and 32.1%, using automated extraction from unstructured and structured data, respectively. Accuracy thresholds (F1 score > 80%) were achieved for all 11 of these data elements by automated extraction from unstructured data. Accuracy thresholds were not met for any of the data elements by automated extraction from structured data.

**Table 1:** Accuracy of automated extraction of data elements

	**Manual annotation**		**EHR structured data using traditional approaches**				**EHR unstructured data using advanced approaches**			
**Features**	**Encounter occurrence**	**Patient-level occurrence**	**Encounter occurrence**	**Recall**	**Precision**	**F1 score**	**Encounter occurrence**	**Recall**	**Precision**	**F1 score**
**Headache**	1,996	1,001	737	33.1%	87.7%	48.1%	1,963	97.8%	91.9%	94.8%
**Migraine**	1,314	809	95	58.5%	82.6%	68.5%	1,233	93.5%	95.1%	94.3%
Headache severity*										
Headache (mild)	104	92	0	0.0%	NA	NA	66	74.0%	81.8%	77.7%
Headache (severe)	236	194	0	0.0%	NA	NA	197	81.1%	65.5%	72.5%
**Severe headache descriptors**										
Throbbing	132	107	0	0.0%	NA	NA	87	75.3%	80.5%	77.8%
Stabbing	53	39	0	0.0%	NA	NA	36	82.1%	88.9%	85.3%
Pulsating	86	65	0	0.0%	NA	NA	53	70.6%	90.6%	79.3%
**Associated symptoms**										
Nausea	1,195	757	39	2.5%	75.0%	4.9%	1,144	95.1%	92.1%	93.6%
Vomiting	886	607	12	1.0%	56.3%	2.0%	864	97.2%	89.2%	93.1%
Photophobia	457	335	0	0.0%	NA	NA	429	86.6%	87.7%	87.1%
Phonophobia	319	232	0	0.0%	NA	NA	281	87.5%	96.8%	91.9%

^*^Sample sizes for moderate headache, disabling headache, debilitating headache, and piercing headache were below requirements for inclusion (< 20 occurrences)

*EHR* Electronic health record, *NA* Not applicable

Among the 2,006 encounters evaluated, migraine outcome model scores based on automated feature extraction of data elements and scoring were an exact match and close match to the manually scored encounter for 77.2% and 82.2% of encounters, respectively. The predefined accuracy threshold of 70% was achieved for both methods of match classification (exact match and close match).

## Discussion

The purpose of this study was to define a scalable framework for outcome validation in disease areas with soft outcomes, such as neurology, for application in RWE generation. Migraine was used as a testbed. The study objectives were to define an outcome model for migraine and to evaluate the accuracy of a technology-driven approach in populating the outcome model using routinely collected data from recent EHR entries at a representative healthcare center.

Innovation in evidence generation is key to improving patient management, enhancing medical decision making, and optimizing healthcare budgets. Clinical trials and prospective observational studies are designed to maximize internal validity and are thus typically limited in size, study population diversity, and duration resulting in a narrow characterization of disease and treatment interventions that limit generalizability of the results [1]. Such a restricted approach to evidence generation cannot support rapid and generalizable inference about disease progression and treatment effectiveness in prevalent disease areas with diverse patient characteristics (such as migraine). As a result, real-world data and RWE are increasingly used to support clinical assertions about the safety and effectiveness of treatments and interventions outside of the clinical trial setting. However, tools used in clinical trials to measure outcomes for disease areas with soft outcomes are often not well suited or specifically designed for analysis of data collected in routine clinical practice, resulting in soft outcome measurements tools only rarely being used by clinicians as decision-making tools with their patients. Development of novel outcome models that capture clinical concepts in the data generated in routine care and management of patients offers a scalable approach to migraine outcome characterization in the real-world setting.

Validity is another important aspect when considering the evidential value of RWE. Advances, including the widespread use of EHRs, increasingly play a key role in the primary collection of large, heterogeneous quantities of medical information [13]; however, in order for clinical assertions to be made based on secondary use of data, the underlying data must be high quality and fit for purpose. High quality is generally defined as data that are accurate, complete, and traceable, while fit for purpose means that the information collected must be appropriate for the research question at hand [14]. In this report and in a previous publication [13], two components of enriching primary data to be high-quality data for secondary use have been addressed: accuracy and traceability. There is no plausible substitute for sampling the data, developing a credible gold standard, and ensuring protocol-required accuracy levels are achieved. The third component, completeness, requires clinical source data such as EHR narratives, and linkage to other data sets such as pharmacy claims, facility claims, and death registries. While highly relevant, the complexity of “completeness” is beyond the scope of the current manuscript. Moreover, while quantification of data may be advocated by some as a pathway to overcome quality challenges, limitations of the available large-scale datasets (including inaccuracy, missing details, and restricted validation options) can easily result in bias and weakness of the ensuing findings. We believe this and other work clearly shows that quantity cannot overcome inherent quality challenges.

In the current study, we relied on enriched data from the unstructured EHR. We have previously conducted a study investigating the accuracy of structured data in examining migraine-related symptoms and concepts and found it lacking [13]. We considered exclusive use of structured data for the outcome model evaluated here but found it similarly lacking in both recall and precision. This is likely due to the increasing use of the problem lists within billing workflow and the strong encouragement by coders to use diseases rather than symptoms to bill [15, 16]. Conversely, automated extraction of migraine symptoms, severity, and descriptors from unstructured data demonstrated accuracy well above the threshold of accurate concept identification. This is believed to be because the use of unstructured data by the clinician reflects the thought process behind diagnosis and treatment and which justifies continued management of the patient by the healthcare provider [17]. Characterizing the accuracy of data and methods used in RWE is a critical step in generating evidence to support clinical assertions.

The two major challenges for study implementation were model development and error stacking. Models are easier to develop in randomized trials since any question can be asked and a research coordinator is available to ensure the correct information is captured. In routinely collected data, the model must rely only on those elements that are typically captured in routine care. The headache specialists who developed this model explored a variety of elements, including headache severity, headache frequency, and associated symptoms. Comparing against elements available in typical documentation, it was found that some elements, such as headache frequency, were simply not captured consistently enough to be used in an outcome model using routine documentation. Furthermore, the migraine outcome model required multiple data elements, with an automated extraction error rate associated with each data element. The accuracy of the migraine outcome model score for a given encounter is dependent on the cumulative error rate of all data elements fed into the model. If each element and attribute have an error rate of 10% and four elements feed into the result for a given encounter, a system would be unlikely to produce the correct migraine outcome score.

We acknowledge several study limitations, which in turn give rise to future areas of research. First, the outcome model was based upon selection of patients likely to have migraine to provide sufficient frequency of disease to make the study feasible; the automated system is unlikely to perform as successfully in patients without migraine and should be applied only to migraine patients. If there is a need identified for characterizing migraine severity and progression in people without a formal migraine diagnosis, the model might need to be revised. Second, our study used a tertiary care data set, and the results generated from this single practice type might not generalize to other healthcare settings. Finally, although language patterns for a clinical domain like migraine tend to be consistent from institution to institution, the accuracy of system performance and model accuracy should be assessed within the healthcare setting and study population of interest to ensure validity in future RWE. It should not be assumed that one technology performing well on unstructured data means other technologies will perform well. In fact, even in this work, natural language processing alone was unable to achieve sufficient extraction accuracy to power a model that met success criteria. AI-based inference-identifying patterns throughout the encounter were required to achieve sufficient element-level accuracy to power the model and meet success criteria. These results cannot be generalized to any technology applied to unstructured data.

## Conclusions

This study outlined an approach to defining and validating an outcome model based on routinely collected data for application in generating RWE, with migraine used as a testbed. We learned that a model using routinely collected data could be built and validated. The outcome model employed here using AI technology applied to unstructured EHR data had high accuracy in generating a migraine outcome score for characterization of disease progression in people living with migraine compared to a migraine outcome score generated by manual annotation. We also learned that the model could be technology enabled; specifically, the model could be accurately populated via computer programming applied to clinical data. Wider implementation of these methods could provide a robust yet flexible approach to credible EHR-based clinical studies. In migraine, the developed model may enable scalable research to support migraine prevention and assist in developing a personalized approach to migraine management.

## Data Availability

The datasets generated and/or analyzed during the current study are not publicly available due to data license restrictions. However, summarized data are available from the corresponding author upon reasonable request.

## Abbreviations

AI: Artificial intelligence
EHR: Electronic health records
NLP: Natural language processing
RWE: Real-world evidence
SQL: Structured query language
